# Progress and Challenges of Immune Checkpoint Inhibitor-Induced Hypophysitis

**DOI:** 10.3390/jcm12103468

**Published:** 2023-05-15

**Authors:** Piaohong Chen, Jianwei Li, Huiwen Tan

**Affiliations:** 1 Department of Endocrinology and Metabolism, West China Hospital, Sichuan University, Chengdu 610041, China; 2 Institute of Pituitary Adenomas and Related Diseases, West China Hospital, Sichuan University, Chengdu 610041, China

**Keywords:** immune checkpoint inhibitors, cytotoxic T lymphocyte antigen, programmed death protein 1, programmed death receptor ligand 1, immune checkpoint inhibitor-induced hypophysitis

## Abstract

Immune checkpoint inhibitors (ICIs) are a new type of antitumor drug which can achieve antitumor goals by blocking the binding of immune checkpoints to their ligands, thereby enhancing the activity of T cells. Meanwhile, ICIs block the binding of immune checkpoints to their ligands, disrupting the immune tolerance of T cells to self-antigens, which may lead to a series of immune-related adverse events (irAEs). Immune checkpoint inhibitor-induced hypophysitis (IH) is a relatively rare irAE. Due to the lack of specificity in clinical manifestations, it is difficult to accurately diagnose IH in a timely manner in clinical practice. However, the risk of adverse events, especially IH, for patients receiving ICIs has not been adequately investigated. Missed or delayed diagnosis may lead to poor prognosis or even adverse clinical outcomes. In this article, we summarize the epidemiology, pathogenesis, clinical manifestations, diagnosis and treatment of IH.

## 1. Definition and Classification of ICIs

Malignant tumors are a major disease that seriously threatens human health. Surgery, radiotherapy and chemotherapy are the main means of tumor treatment. Targeted therapy and immunotherapy are new methods of tumor treatment that have been developed in recent years and are revolutionary breakthroughs in tumor treatment. Among them, immune checkpoint inhibitors (ICIs) are the main methods of current immunotherapy. Immune checkpoints are one normal part of the immune system. ICIs work by blocking checkpoint proteins from binding with their partner proteins. However, ICIs lack tumor tissue specificity and destroy the immune tolerance of T cells to self-antigens, which can lead to a series of immune-related adverse events (irAEs) [1]. As a common endocrine irAE, immune checkpoint inhibitor-induced hypophysitis (IH) cannot be diagnosed in a timely and accurate manner because of its lack of specificity in clinical manifestations, such as anterior pituitary insufficiency. Therefore, clinicians need to be vigilant enough in the process of tumor immunotherapy. On the one hand, the diagnosis and treatment of IH in a timely manner can avoid pituitary crises or even life-threatening situations. On the other hand, misdiagnosis of IH may lead to clinical decisions to discontinue immunotherapy, which may lead to tumor progression [2]. IH has brought new challenges to tumor treatment. Therefore, in this review, we summarize the epidemiology, pathogenesis, clinical manifestations, diagnosis and treatment of IH.

Immune checkpoints are small molecules expressed on the surface of T lymphocytes and play an important role in maintaining immune homeostasis and autoimmune tolerance.

There are two broad categories of immune checkpoints. One type of immune checkpoint, such as CD28 and CD27, enhances T-cell activity by mediating stimulatory signals; the other type of immune checkpoint mainly includes cytotoxic T-lymphocyte antigen 4 (CTLA-4) and programmed death protein 1 (PD-1), which blunts T cells by transmitting inhibitory signals and plays an important role in immune tolerance.

CTLA-4 acts at the initial stage of the immune response and can transmit T-cell inhibitory signals after binding to its ligands CD80 and CD86 [3]. In contrast, PD-1 generally acts in the later stage of the immune response and inhibits the activity and immune function of T cells after binding to its ligands’ programmed death receptor ligand 1/2 (PD-L1/2) [4,5]. Tumor cells evade the body’s immune attack by enhancing the inhibitory signals of CTLA-4 and PD-1 [3]. ICIs are a class of specific IgG antibodies that recognize the inhibitory immune checkpoints CTLA-4 or PD-1 [6] and immune checkpoint ligand PD-L1/2, which restore or enhance the killing power of T cells against tumor cells by blocking the interaction with their ligands to achieve antitumor effects [7]. Currently, ICIs mainly include CTLA-4 inhibitors and PD-1/PD-L1 inhibitors. To date, two CTLA-4 inhibitors, ipilimumab and tremelimumab, have been approved for clinical use in the European Union and the Food and Drug Administration (FDA) has approved them in the U.S., mainly for the treatment of melanoma. The PD-1 inhibitors that have been approved mainly include pembrolizumab, nivolumab and cemiplimab, which are mainly used for the treatment of malignant tumors such as melanoma, non-small-cell lung cancer (NSCLC), renal cancer, Hodgkin lymphoma, head and neck squamous cell carcinoma and urothelial carcinoma. There are also four types of PD-1 inhibitors developed independently, namely toripalimab, sintilimab, camrelizumab and tislelizumab, all of which have been approved by the State Food and Drug Administration (SFDA) in China and are mainly approved for the treatment of Hodgkin lymphoma. PD-L1 inhibitors mainly include atezolizumab, avelumab and durvalumab, which are mainly used for the treatment of urothelial cancer and NSCLC. To expand the clinical indications of the above ICIs for more tumors, many preclinical studies are underway.

Accessed on 30 June 2022, there are a total of 616 ongoing clinical studies of CTLA-4 inhibitors and a total of 4890 ongoing clinical studies of PD-1/PD-L1 inhibitors registered in the U.S. National Library of Medicine’s official website of clinical trials (https://clinicaltrials.gov/, accessed on 30 June 2022) and China’s drug clinical trial information and publicity platform (http://www.chinadrugtrials.org.cn, accessed on 30 June 2022). It is expected that the clinical indications of these two types of ICIs will further increase in the near future, and the clinical application will become increasingly extensive. The representative drugs of CTLA-4 monoclonal antibody and PD-1/PD-L1 monoclonal antibody that have been approved for marketing, their time of approval and their detailed clinical indications are shown in Table 1.

## 2. Overview of irAEs

ICIs disrupt the maintenance of the body’s autoimmune tolerance, leading to a series of irAEs through multiple pathways, including autoreactive T cells, autoantibodies and cytokines. IrAEs can involve multiple organ systems, most commonly in the skin and gastrointestinal tract, followed by the lungs, blood, urinary, nervous and musculoskeletal systems, as well as cardiovascular systems [8]. The endocrine system is one of the most easily involved systems by irAEs due to its rich blood supply [9].

Since the European Society for Medical Oncology (ESMO) [10] published the world’s first clinical practice guideline on the management of irAEs in 2017, the American Society for Immunotherapy [8], the American Society of Clinical Oncology (ASCO), the National Comprehensive Cancer Network (NCCN) [11] and the Chinese Society of Clinical Oncology (CSCO) have successively issued clinical guidelines for the management of irAEs.

Since 2018, the British Endocrine Society [12], the Japanese Endocrine Society [13] and the French Endocrine Society [14] have successively published guidelines for the management of adverse endocrine reactions related to ICIs therapy. In 2021, The Chinese Society of Endocrinology (CSE) and the Professional Committee of Oncology and Endocrinology of the Chinese Anti-Cancer Association will also successively publish two expert consensuses on irAEs of the endocrine system caused by ICIs [15,16]. Endocrine-system-related irAEs have been reported thus far, including immunotherapy-related diabetes, thyroid dysfunction, adrenal insufficiency and IH [17]. Although the overall incidence of IH is not high, it has gradually attracted the attention of clinicians and scholars all over the world [18,19,20]. Due to IH’s lack of specificity in clinical manifestations, misdiagnosis and delayed diagnosis lead to poor prognosis for patients.

The abovementioned expert consensus and clinical guidelines on irAEs of the endocrine system have explained the diagnosis and treatment of IH. Correct diagnosis and timely treatment of IH is very important for the prognosis of tumor patients.

## 3. Epidemiology of ICI-Induced Hypophysitis

The first case of IH was reported by Phan et al. in 2003 [21]. A 54-year-old man with melanoma developed personality changes and memory loss after four cycles of treatment with the CTLA-4 inhibitor ipilimumab. During the 5th cycle of treatment, laboratory tests showed that the patient’s anterior pituitary dysfunction, thyroid-stimulating hormone (TSH), free thyroxine (FT4), adrenocorticotrophic hormone (ACTH), cortisol, growth hormone (GH), prolactin (PRL) and testosterone (T) levels were all undetectable. His saddle MRI showed a plump pituitary gland. The patient was diagnosed with IH, and his clinical symptoms improved following hydrocortisone, thyroxine and testosterone replacement therapy.

In 2016, Ishikawa et al. [22] reported a case of PD-1 inhibitor-related IH in a 55-year-old male Japanese patient with melanoma and right adrenal metastasis. The patient developed nausea, anorexia and weight loss after four cycles of nivolumab treatment. Laboratory tests showed that ACTH and cortisol decreased significantly, and the symptoms gradually improved after hydrocortisone treatment. Therefore, the patient was diagnosed with nivolumab-related IH. Since then, IH has gradually been reported and has received more attention from clinicians [17,23,24].

IH is a common endocrine-system-related irAE. The incidence of IH is related to the type and treatment regimen of ICI [25]. To date, multiple systematic reviews have conducted statistical analysis on the incidence of IH [2,18,19,20,26,27,28,29,30,31,32,33], and the results show that the combination of CTLA-4 inhibitors and PD-1/PD-L1 inhibitors has the highest incidence of IH. It constitutes up to 10% of patients who received treatment [31]. The incidence of IH associated with CTLA-4 inhibitor and PD-1/PD-L1 inhibitor monotherapy is much lower than that of combination therapy, accounting for 5% and 1%, respectively [26,29]. A summary of IH reports in ICI-related adverse reactions is shown in Table 2.

With the development of ICI clinical trials and increasing clinical application, the incidence statistics of IH may change. The incidence of IH associated with the CTLA-4 inhibitor ipilimumab has been reported to increase with increasing drug dose [34], and the incidence of IH was approximately 1.8–3.3% in patients receiving low-dose ipilimumab (<3 mg/kg). When the ipilimumab dose was increased (>3 mg/kg), the incidence of IH reached 9–17% [9]. Currently, there are few reports on PD-1/PD-L1 inhibitor-related IH, so the relationship between the incidence of PD-1/PD-L1 inhibitor-related IH and dose is still inconclusive. Although studies have shown no gender difference in irAEs [35], CTLA-4 inhibitor-related IH mostly occurs in men around the age of 60 [4], with a male-to-female ratio of about 4:1 [36]. Male predominance may be because the CTLA-4 inhibitor ipilimumab is commonly used in the treatment of melanoma, and men have a higher incidence of melanoma than women [25]. The median age of onset of PD-1/PD-L1 inhibitor-related IH is between 60 and 65 years old, and the male-to-female ratio is about 1–3:1 [37,38]. The data were derived from two follow-up studies involving 17 and 22 patients with PD-1 inhibitor-induced IH. Due to the limited number of patients in the two studies, whether there is a sex difference in PD-1/PD-L1 inhibitor-related IH still needs to be further supported by clinical data. Recent studies have found that the occurrence time of IH is related to the type of ICI [37,38,39,40]. IH associated with combination therapy of CTLA-4 inhibitor and PD-1/PD-L1 inhibitor generally occurs approximately 4 weeks after treatment. CTLA-4 inhibitor monotherapy mostly occurs approximately 9.3 weeks after the initial treatment [38]. PD-1/PD-L1 inhibitor-related IH occurs relatively late, with a median onset time of approximately 28 weeks [37], and IH has even been reported to occur approximately 60 weeks after discontinuation of PD-1/PD-L1 inhibitors [41].

In conclusion, male patients currently receiving CTLA-4 inhibitor and PD-1/PD-L1 inhibitor combination therapy have the highest incidence of IH, followed by CTLA-4 inhibitor monotherapy, and there may be a dose-dependent relationship. The lowest incidence was in patients receiving PD-1/PD-L1 inhibitor monotherapy. The onset time ranged from 4 weeks to 28 weeks after receiving ICI treatment, and some even set on after the drug was discontinued. The relevant epidemiological data of IH still need to be supplemented and improved by more clinical studies in the future.

## 4. Pathogenesis of ICI-Induced Hypophysitis

The exact pathogenesis that induces irAEs is still not clear. It was believed that its pathophysiological mechanism is related to the imbalance of immune checkpoints in maintaining immune system homeostasis. At present, some progress has been made in research on the mechanism of IH. Iwama et al. [42] confirmed the expression of CTLA-4 antigen in the pituitary tissue of a mouse hypophysitis model induced by a CTLA-4 inhibitor in animal experiments. An autopsy of patients who died after IH revealed that their pituitary tissue expressed CTLA-4, especially on pituitary TSH and PRL cells [42,43], suggesting that CTLA-4 inhibitors can directly bind to CTLA-4 on pituitary cells and degrade it as an antigen presented to CD8^+^ T cells, eventually inducing IH through the type IV hypersensitivity system [44]. This is consistent with the clinical manifestations that CTLA-4 inhibitor-related IH is prone to impaired thyroid axis function. In addition, among CTLA-4 inhibitors, the IgG subtype of ipilimumab is IgG1, while the IgG subtype of tremelimumab is IgG2 [42]. Both IgG subtypes can trigger antibody-dependent cell-mediated cytotoxicity (ADCC) and the classical complement activation pathway of type II hypersensitivity reactions [45]. Thus, CTLA-4 inhibitors can induce IH through type II hypersensitivity reactions.

Compared with CTLA-4 inhibitors, the mechanism of PD-1/PD-L1 inhibitor-induced IH is still unknown. Among them, PD-LI inhibitors are also of the IgG1 subtype, but their Fc fragments are modified, so they cannot induce IH through type II hypersensitivity reactions [46]. Since the main components of PD-1 inhibitors are IgG4-like IgG-4κ or IgG4 monoclonal antibodies, Chinese scholars speculate that PD-1 inhibitor-related IH may be similar to the pathogenesis of IgG4-related hypophysitis [47]. However, in fact, the specific pathogenesis of IgG4-related hypophysitis still has not been fully elucidated [48]. Antipituitary antibodies are present in the serum of patients with IgG4-related hypophysitis. The researchers treated the biopsied pituitary tissue with these antipituitary antibodies and found that growth hormone and opioid melanin may be the suspected self-antigens [49,50]. Keitaro Kanie et al. [51] used a similar method to process serum and tumor specimens from 20 patients with IH after treatment with PD-1/PD-L1 inhibitors. The results show the presence of antipituitary antibodies, anticorticotropin antibodies and anti-GH antibodies in the peripheral blood of these IH patients. There are antipituitary antibodies or antipituitary hormone autoantibodies both in the peripheral blood of patients with IgG4-related hypophysitis and PD-1/PD-L1 inhibitor-related IH, suggesting that PD-1/PD-L1 inhibitor-related IH and IgG4-related hypophysitis may have similar pathogenesis. Autoimmunity is involved, but the specific pathogenesis needs to be further explored. In addition, Tahir et al. [52] used the serological analysis of a recombinantly expressed cDNA clone (SEREX) to screen for autoantibodies in serum samples from patients with IH induced by the CTLA-4 inhibitor ipilimumab, the PD-1 inhibitor nivolumab, or a combination of two ICIs, which revealed that antiguanine nucleotide-binding protein G subunit alpha (GNAL) and anti-integral membrane protein 2B (ITM2B) autoantibodies were significantly increased. GNAL and ITM2B are proteins expressed in normal brain tissue [53,54]. Therefore, it is speculated that anti-GNAL and anti-ITM2B antibodies may be potential risk factors for the occurrence of IH [52].

In short, CTLA-4 inhibitors induce IH mainly through type II and type IV hypersensitivity reactions, and the pathogenesis of PD-1/PD-L1 inhibitor-related IH may be similar to IgG4-related hypophysitis. Anti-GNAL and anti-ITM2B antibodies may also potentially be involved in IH. However, the exact pathogenesis of IH remains to be further explored.

## 5. Clinical Features of ICI-Induced Hypophysitis

The clinical presentation of patients with IH often lacks specificity. In general, headache, fatigue, loss of appetite, and an enlarged pituitary gland are the main features, while central diabetes insipidus and visual disturbances are relatively rare. There are certain differences in specific performance due to different types of ICIs [44].

CTLA-4 inhibitor-related IH commonly manifests as headache and fatigue [38], and panpituitarism is often accompanied by pituitary enlargement [38]. IH associated with PD-1/PD-L1 inhibitors often presents with fatigue and poor appetite [37] and is often characterized by isolated secondary adrenal insufficiency [37,55]. According to the literature reports, the two types of ICI-related IH have different degrees of involvement in anterior pituitary function. The most common presentations of CTLA-4 inhibitor-related IH are FSH and/or LH deficiency (88%) and TSH deficiency (81%), followed by ACTH deficiency (55%) [38]. PD-1/PD-L1 inhibitor-related IH has ACTH deficiency in nearly 100% of cases [37], while TSH, GH and FSH/LH deficiency are less common, and its incidences were 11.8%, 13.3% and 18.8% of the treated population, respectively [37]. Most important of all is that the clinical manifestations of ACTH deficiency sometimes are the most severe [23,56,57]. Severe IH patients may present with high fever, low blood pressure, electrolyte disturbances (mainly hyponatremia) and even pituitary crises, such as disturbance of consciousness and coma. Since the symptoms of IH are not specific, they often need to be identified with sepsis with similar manifestations [15]. Central diabetes insipidus due to posterior pituitary involvement in IH is rare. To date, only a few reports of central diabetes insipidus have been reported. Among them, two cases were caused by the CTLA-4 inhibitor ipilimumab [58,59], two cases were caused by the PD-1 inhibitor nivolumab [60,61], and one case was caused by the PD-L1 inhibitor avelumab [62]. Notably, this type of IH-related hyponatremia needs to be differentiated from the syndrome of inappropriate antidiuretic hormone secretion (SIADH) caused by the tumor.

Pituitary or sellar imaging is helpful for differentiating IH from pituitary tumor metastases, pituitary adenomas and infectious pituitary diseases. Among them, pituitary-enhanced MRI is a more sensitive method. In total, 98% of patients with CTLA-4 inhibitor-related IH had pituitary enlargement [38], while only 28% of patients with PD-1/PD-L1 inhibitor-related IH developed pituitary enlargement [38]. A longitudinal retrospective study suggested that imaging changes in the pituitary can occur before or after the onset of clinical symptoms and changes in hormone levels [36]. Therefore, the absence of significant pituitary growth in early pituitary-enhanced MRI does not rule out IH [4,23]. The differences in clinical manifestations, laboratory tests and pituitary MRI between CTLA-4 inhibitor and PD-1/PD-L1 inhibitor-related IH are summarized in Table 3.

**Figure 1:**
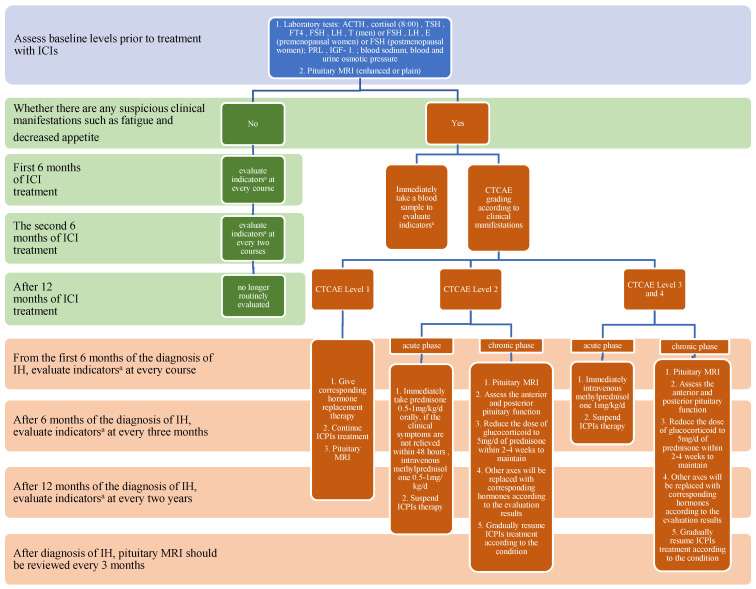
The diagnosis, treatment and follow-up process of ICI-induced hypophysitis. Note: ^a^: laboratory tests; ACTH, cortisol (8:00), TSH, FT4, FSH, LH and T (men) or FSH, LH and E (premenopausal women) or FSH (postmenopausal women); PRL, IGF-1.; blood sodium, blood and urine osmotic pressure.

The symptoms of IH present with anterior pituitary hypofunction and imaging changes in the pituitary, which lack clinical specificity. Therefore, IH is easily missed or delayed in diagnosis. However, laboratory tests are sensitive and useful for diagnosing IH. Castinetti and Albarel et al. [63,64] suggested that pituitary-related hormones and pituitary MRI should be evaluated before using ICIs. Pituitary-related hormones include TSH, FT3 and FT4 in the hypothalamic–pituitary–thyroid axis (HPT axis), ACTH and cortisol in the hypothalamic–pituitary–adrenal axis (HPA axis), as well as ACTH stimulation test if necessary, and FSH, LH and T/E2 in the hypothalamic–pituitary–gonad axis (HPG axis). In addition, assessments of GH, IGF-1 and PRL are also useful. Meanwhile, indicators such as serum electrolytes, blood osmolality, urine osmolality and urine-specific gravity need to be measured to evaluate posterior pituitary function. According to the median onset time of IH, ClaireBriet et al. [39] suggested that during the first 6 months of ICI treatment, anterior pituitary function and blood electrolytes should be tested in each course of treatment for patients without symptoms of hypopituitarism. During the second 6 months of treatment, assessments are recommended every 2 courses. After 12 months of treatment with ICIs, pituitary hormone assessments are no longer routinely performed, because the usual time of onset of IH has passed (Figure 1). However, if the patient develops suspicious symptoms such as headache, fatigue, loss of appetite, etc., it is still necessary to evaluate anterior pituitary function and blood electrolytes. Pituitary imaging is not required in the assessment of anterior pituitary function described above; however, if anterior pituitary hypofunction is present, pituitary imaging is needed. Therefore, in the process of using ICIs, if the patient has clinical symptoms of suspected anterior pituitary hypofunction, anterior and posterior pituitary function should be evaluated immediately, and pituitary imaging examination should be completed at the same time [56].

## 6. Diagnosis of ICI-Induced Hypophysitis

At present, there is no consistent diagnostic standard for IH in the guidelines of various countries or regions around the world [14,39], but they all recommend that when patients using ICIs have clinical symptoms of suspected anterior pituitary dysfunction (such as fatigue, decreased appetite, etc.) and/or hyponatremia and/or examination suggests ≥1 anterior pituitary hormone deficiency (essential TSH or ACTH deficiency) and/or abnormal pituitary imaging findings, IH should be considered. In 2021, the expert consensus of the Chinese Society of Endocrinology and the Professional Committee of Oncology and Endocrinology of the Chinese Anti-Cancer Association recommended that the diagnostic criteria for IH be that patients develop more than one anterior pituitary hormone deficiency (must have TSH or ACTH deficiency) after using ICIs, accompanied by abnormal pituitary MRI performance or that patients develop more than two anterior pituitary hormone deficiencies (must have TSH or ACTH deficiency) and headache, as well as other symptoms of anterior pituitary hypofunction [15,16]. However, this criterion may fail to diagnose some patients with PD-1/PD-L1 inhibitor-related IH, whose main manifestation is isolated hypoadrenalism without pituitary MRI abnormalities [38]. Therefore, special vigilance should be paid to such patients without pituitary MRI abnormalities.

The severity of IH is different, and the follow-up diagnosis and treatment are also different. The “CSCO management guidelines on toxicities from immune checkpoint inhibitors” issued by the Chinese Society of Clinical Oncology (CSCO), as well as the “Chinese expert consensus on the immune checkpoint inhibitors-induced endocrine immune-related adverse events (2020)” issued by the Immunological Endocrinology Group, Endocrinology Society and Chinese Medical Association [15], all recommended the common terminology criteria for adverse events (CTCAE) published by the National Cancer Institute (NCI) to describe the severity of IH.

The severity of IH is graded from 1 to 5. Grade 1 means mild (no symptoms or mild symptoms), and Grade 2 means moderate (mild-to-moderate and mild local symptoms; noninvasive treatment needed; mild impairment of self-care; and no limitation of daily activities). Grade 3 means severe (severe or clinically important, but not temporarily life-threatening; requiring or prolonged hospitalization; severely impaired self-care; and affecting daily activities). Grade 4 means life-threatening (life-threatening, requiring urgent intervention; unable to perform daily activities). Grade 5 means death (Table 4). At present, IHs of CTCAE grades 1 to 5 have been reported, but most of them are CTCAE grades 1 to 2. Severe IHs (CTCAE grades 3 to 5) are relatively rare, accounting for approximately 10% to 40% of all cases of IH [18,19,20].

## 7. Treatment and Prognosis of ICI-Induced Hypophysitis

For the treatment and prognosis of IH, the British Endocrine Society [12], the Chinese Endocrinology Society [15] and the Chinese Anti-Cancer Association Tumor Endocrinology Professional Committee [16] all recommended that the severity of IH should be graded by CTCAE, according to the clinical manifestations of patients. Meanwhile, laboratory tests, such as anterior pituitary hormones, blood electrolytes, blood osmolality, urine osmolality and urine specific gravity, are necessary.

For IH patients assessed as CTCAE Grade 1 (mild fatigue, anorexia or mood changes without headache or asymptomatic), the ESMO and Chinese expert consensus suggests that ICI treatment can be continued. At the same time, according to the results of laboratory evaluation, the corresponding hormone replacement therapy should be performed for the damaged pituitary-target gland axis [16,65]. For CTCAE Grade 2 IH patients (headache without visual disturbance), ESMO recommends that ICI therapy should be suspended, and prednisone at 0.5–1 mg/kg/d should be administered orally [65]. If the patient’s symptoms do not relieve within 48 h, intravenous methylprednisolone at 0.5–1 mg/kg/d should be given, and the dose of prednisone should be reduced to 5 mg/d oral maintenance therapy within 2–4 weeks [65]. The damaged pituitary-target gland axis should be treated with corresponding hormone replacement therapy. Since discontinuation of ICIs does not affect the natural history of hypophysitis [66], and the survival benefit of continuation of ICI treatment of tumors is far greater than the risk of anterior pituitary dysfunction, ESMO recommends that patients with IH continue to use ICIs after the acute phase [65]. The expert consensus of the Chinese Cancer Society [15,16] proposed that ICI treatment should not be interrupted for CTCAE Grade 2 IH. For patients with IH in CTCAE Grades 3–4 (severe mass effect or severe headache; visual disturbance; or severe adrenal insufficiency or hypotension; or severe electrolyte disturbance), all guidelines recommend suspending ICI therapy [67] and immediately starting intravenous methylprednisolone at 1 mg/kg/d, as well as reducing glucocorticoids to prednisone at 5 mg/d oral maintenance therapy within 2–4 weeks. At the same time, according to the test results, other damaged pituitary–target gland axes were given corresponding hormone replacement therapy. Until the acute phase of IH is relieved, ICI treatment is continued [65]. For patients with ACTH and TSH deficiency, the Japan Endocrine Society [13] recommends that low-dose levothyroxine (12.5–25 µg/d) be administered after hydrocortisone (10–20 mg/d) for 5–7 d, adjusting the levothyroxine dose according to the serum level of FT4 to avoid inducing iatrogenic adrenal crisis. Studies have found that receiving high-dose (average daily prednisone dose greater than 7.5 mg) glucocorticoid therapy will affect the clinical antitumor efficacy of ICIs, reduce the overall survival rate of patients [68], increase the risk of infection, hyperglycemia, etc. [66], and cannot significantly improve the prognosis of anterior pituitary dysfunction [11]. In summary, high-dose glucocorticoid therapy is not recommended [13,15]. However, for patients with intractable headache and/or visual impairment, the Japanese Endocrine Society recommends the use of prednisone at 0.5–1.0 mg/kg/d [13]. Once symptoms such as intractable headaches and/or visual disturbances resolve, glucocorticoids should be rapidly reduced to physiologic replacement doses within 2–4 weeks [65]. Since HPG axis damage is easier to recover and poses a lower threat to the survival of patients, the French Endocrine Association [14] suggested that patients with HPG axis damage should be followed up for three months. If the HPG axis has not recovered after three months, the corresponding sex hormone replacement therapy can be given according to the patient’s age and other circumstances. For patients with an impaired GH/IGF-1 axis, the French Society of Endocrinology does not recommend GH replacement therapy due to the background of the patient’s primary malignancy [14]. If the posterior pituitary is damaged and diabetes insipidus occurs, the Chinese Society of Endocrinology recommends the use of synthetic antidiuretic hormone (ADH) for treatment [15]. Based on the recommendations of many guidelines, this review article briefly summarizes the diagnosis and treatment process of IH (Figure 1).

Compared with other irAEs, the prognosis of IH is relatively good. Generally, after the acute phase of the disease, the treatment of ICIs can be resumed. Permanent HPA axis damage occurs in 86–100% of IH patients, requiring long-term glucocorticoid replacement therapy [69]. Permanent HPA axis damage in PD-1/PD-L1 inhibitor-related IH is more common [37]. Today, with the increasing use of PD-1/PD-L1, it is especially necessary to attract the attention of clinicians. In addition, studies have shown that the HPT axis often recovers at approximately 10.5 weeks, and the HPG axis can recover at approximately 25 weeks [66]. However, 13–36% of patients may have permanent damage to the HPG axis [66,69]. Since the degree of damage and the recovery time to the abovementioned pituitary-target gland axis is not synchronized [15], the French Endocrine Society [14] recommends that from the first 6 months of the diagnosis of IH, the pituitary-target gland axis hormones need to be evaluated before each course of treatment to facilitate the timely detection of new pituitary-target gland axis damage. After 6 months, the pituitary-target gland axis hormones were evaluated every 3 months. After 12 months, the pituitary-target gland axis hormones were evaluated twice per year. Meanwhile, it is recommended to review the pituitary MRI every 3 months after the diagnosis of IH, which can evaluate the progress of IH and help rule out pituitary metastases from the primary tumor (Figure 1).

## 8. Summary

In recent years, rapid progress has been made in the application of ICIs in patients with advanced malignant tumors. IH has received increasing attention as a common endocrine irAE. On the one hand, the clinical manifestations of IH lack specificity. On the other hand, anterior pituitary insufficiency can threaten the safety of patients. Interrupting ICI therapy can lead to tumor progression. Therefore, early and correct diagnosis of IH is particularly important. The pathogenesis of IH caused by CTLA-4 inhibitors and PD-1/PD-L1 inhibitors is different. CTLA-4 inhibitor-induced IH is often accompanied by total anterior pituitary hypofunction combined with pituitary enlargement, while PD-1/PD-L1 inhibitor-induced IH is mainly characterized by isolated ACTH deficiency. Assessment of anterior pituitary hormones, especially the HPA axis, is one of the more sensitive methods for diagnosing IH. Dynamic monitoring of pituitary-target gland axis hormones and pituitary MRI is necessary before and during the use of ICIs. Once IH has been diagnosed, hormone replacement therapy should be started immediately, and high-dose glucocorticoid pulse therapy should be used with caution. It should be evaluated whether to suspend ICIs treatment based on the risk of benefit and harm. Timely and accurate diagnosis and treatment of IH has important clinical significance for ensuring patient safety and improving patient prognosis.

## Figures and Tables

**Table 1:** Representative drugs and indications of CTLA-4 inhibitors and PD-1/PD-L1 inhibitors.

Drug Information	CTLA-4 Inhibitors		PD-1 Inhibitors							PD-L1 Inhibitors		
	Ipilimumab	Tremelimumab	Nivolumab	Pembrolizumab	Cemiplimab	Toripalimab	Sintilimab	Camrelizumab	Tislelizumab	Atezolizumab	Avelumab	Durvalumab
Drug name	Yervoy	No brand	Opdivo	Keytruda	Libtayo	Tuoyi	Daboshu	Erica	Baizean	Tecentriq	Bavencio	Imfinzi
IgG type	IgG1	IgG2	IgG4	IgG4	IgG4	IgG4	IgG4	IgG4k	IgG4	IgG1k	IgG1	IgG1k
Main indication	melanoma	Hepatocellular cancer, malignant mesothelioma	Melanoma, Esophageal squamous cell cancer and other tumors ^a^	Melanoma, metastatic NSCLC and other tumors ^b^	CSCC	Melanoma, nasopharyngeal cancer	Classical Hodgkin’s lymphoma	Classical Hodgkin’s lymphoma	Classical Hodgkin’s lymphoma, urothelial cancer	Urothelial cancer, NSCLC	Merkel cell cancer, urothelial cancer	Urothelial cancer, NSCLC
Number of ongoing trials	495	121	1088	1425	80	291	264	301	244	557	167	473

Note: NSCLC, non-small-cell lung cancer; CSCC, squamous cell carcinoma of the skin. ^a^: NSCLC; CSCC; renal cell cancer; small cell lung cancer; classical Hodgkin’s lymphoma; urothelial cancer; head and neck Squamous cancer; microsatellite instability–high/deficient mismatch repair cancer; colorectal cancer; hepatocellular cancer. ^b^: classical Hodgkin’s lymphoma; large B-cell lymphoma; Merkel cell cancer; head and neck squamous cell cancer; urothelial cancer; microsatellite instability–high/deficient mismatch repair cancer; gastric cancer; cervical cancer; hepatocellular cancer.

**Table 2:** Incidence of ICI-induced hypophysitis reported in the literature from 2017 to the present.

Author	CTLA-4 Inhibitors Combination PD-1/PD-L1 Inhibitors		CTLA-4 Inhibitors		PD-1/PD-L1 Inhibitors	
	CTCAE1 to 5	CTCAE3 to 5	CTCAE1 to 5	C TCAE3 to 5	CTCAE1 to 5	CTCAE3 to 5
Barroso-Sousa et al. [18]	6.4% (Ipi + Niv)	NA	3.2%	NA	0.5%	NA
El Osta et al. [26]	8.0%	1.7%	5.40%	3.3%	0.30%	0.1%
Wang et al. [27]	NA	NA	NA	NA	0.85%	0.59%
Baxi et al. [28].	NA	NA	NA	NA	0.3%	0.2%
De Filette et al. [29].	8.8% (Ipi + Niv), 10.5% (Ipi + Pem)	NA	1.8%(Tre), 5.6% (Ipi)	NA	0.5% (Niv), 1.1%(Pem)	NA
Xu et al. [30].	NA	NA	3.3%	1.7%	NA	NA
Wang et al. [2]	NA	2.0%	NA	5.0%	NA	1%
Zhang et al. [19]	9.7%		NA	NA	NA	NA
Almutairi et al. [31]	10.4% (Ipi + Niv), 10.46% (Ipi + Pem)	2.48% (Ipi + Niv)	4.13% (Ipi)	1.21% (Ipi)	0.31% (Niv), 0.66%(Pem)	0.15% (Niv), 0.68% (Pem)
Lu et al. [20]	7.68%	1.66%	4.53%	0.78%	0.41%	0.06%
Xing et al. [32]	8.38% (Ipi + Niv)	2.48% (Ipi + Niv)	NA	NA	0.47% (Niv)	0.35% (Niv)
Li et al. [33]	10%	1.1%	4.2%	1.7%	0.6%	1.5%

Note: CTCAE, the common terminology criteria for adverse events; Ipi, ipilimumab; Niv, nivolumab; Pem, pembrolizumab; Tre, tremelimumab; Atz, atezolizumab; Ave, avelumab; NA, not available.

**Table 3:** Differences between the two types of IH.

Clinical Manifestations	CTLA-4 Inhibitors-Induced Hypophysitis	PD-1/PD-L1 Inhibitors-Induced Hypophysitis
The median onset age	60 years	60~65 years
Gender difference	Male: female 4:1	Male: female 1:1~3:1
The median onset time	9.3 weeks	28 weeks
Clinical manifestations	Headache and fatigue as the first manifestation, accompanied by visual impairment, hypothyroidism, loss of libido (hypohypophysis).	Fatigue and loss of appetite as the first manifestation, accompanied by weight loss, hypotension, etc. (isolated ACTH deficiency)
Laboratory tests	ACTH deficiency 55%; TSH deficiency 81%; FSH/LH deficiency 88%; Hyponatremia 45%; 12.5% lower IGF—112.5%;	ACTH deficiency of 100%; TSH deficiency 11.8%; FSH/LH deficiency 18.8%; Hyponatremia 52.9%; lower IGF—113.3%;
Pituitary MRI	pituitary enlargement, stalk enlargement, or enhancement changes after injection of contrast agent often occur, an incidence of 98%	pituitary enlargement rate 28%

Note: ACTH, adrenocorticotropic hormone; TSH, thyroid stimulating hormone; FSH, follicle-stimulating hormone; LH, luteinizing hormone; IGF-1, insulin-like growth factor 1.

**Table 4:** CTCAE grading and specific clinical manifestations of ICI-induced hypophysitis.

Grade	Description of CTCAE	Clinical Manifestations
Level 1	Mild: Asymptomatic or mildly symptomatic.	Mild fatigue or anorexia, without headache or asymptomatic.
Level 2	Moderate: Mild-to-moderate, mild local symptoms; noninvasive treatment needed; mild impairment of self-care; no limitation of daily activities.	Headache without visual disturbances or fatigue, mood changes without hemodynamic instability and electrolyte disturbances.
Level 3	Severe: Severe or clinically important but not temporarily life-threatening; requiring hospitalization or prolonged hospitalization; severely impaired self-care ability; affecting daily activities.	Headache with visual disturbances, or adrenal insufficiency with hypotension, electrolyte disturbances.
Level 4	Life-Threatening: Life-threatening, requiring urgent intervention; inability to perform daily activities.	Severe headache with visual disturbance, or severe adrenal insufficiency with shock, severe electrolyte.
Level 5	Death.	Death.

Note: CTCAE, the common terminology criteria for adverse events.

## Data Availability

Data Availability Statement:No new data were created.
